# Segmental resection combined with anoplasty for the treatment of circumferential mixed hemorrhoids

**DOI:** 10.1590/1414-431X20198102

**Published:** 2019-05-16

**Authors:** Jing Wu, Keqiang Yu, Changyao Lv, Wenzhu Lu, Hongbo He

**Affiliations:** Department of Integrated Traditional Chinese and Western Medicine, West China Hospital of Sichuan University, Chengdu, China

**Keywords:** Circumferential mixed hemorrhoids, Segmented and plastic hemorrhoidectomy, Anal function, Anal smoothness, Complications

## Abstract

Circumferential mixed hemorrhoids are very difficult to treat non-surgically. Therefore, it is important to explore the surgical methods for its complete resolution as well as maintenance of normal anal anatomy and function. The present study was designed to evaluate the effect of segmented and plastic hemorrhoidectomy (SPH) on patients with circumferential mixed hemorrhoids. A total of 300 patients with circumferential mixed hemorrhoids were divided into experimental group (n=150) undergoing SPH and control group (n=150) undergoing Milligan-Morgan hemorrhoidectomy. There were no differences in cure and effectiveness rates between two groups. Compared with the control group, patients in the experimental group had shorter healing time (15.7±1.3 *vs* 12.5±0.7 days) and recovery to normal activity (18.5±2.7 *vs* 14.7±1.2 days). In addition, anal function of all patients in the experimental group was normal during short- and long-term follow-up. However, more cases in the control group showed anal dampness and itching, and poor control of intestinal liquid. Compared with the control group, patients in the experimental group had better outcomes in overall anal function and smoothness at 6, 12, and 18 months after operation as well as patient satisfaction. Furthermore, the rating in the visual analogue scale for defecation pain and edema in the experimental group was less than that in the control group. SPH was more effective, had fewer complications, better protection of anal function, and a better cosmetic result.

## Introduction

Hemorrhoids are cushions of submucosal tissue containing venules, arterioles, and smooth muscle fibers (1,2). Hemorrhoids are common in both males and females (3). Circumferential mixed hemorrhoids are very difficult to treat non-surgically (4). Clinical symptoms include circumferential prolapse, repeated bleeding, local dampness, and itching, and pathologic changes include local tissue fibrosis, disappearance of the anal dentate line, and downward movement of the anus and anal cushion (5,6). Surgical methods for mixed hemorrhoids include the classic Milligan-Morgan hemorrhoidectomy and the procedure for prolapse and hemorrhoids (PPH) (2,7). Although the Milligan-Morgan hemorrhoidectomy is simple, effective, and the gold standard for mixed hemorrhoid surgery, it has the disadvantages of scar formation, fibrotic stenosis, post-operative pain, bleeding, slow healing, and slow recovery for many patients (8). PPH surgery was developed by the Italian, A. Longo (9). PPH surgery is mainly indicated for grade II-III internal hemorrhoids (8) and has the advantages of less post-operative pain, less bleeding, a shorter hospitalization time, and a shorter recovery time (10–12); however, PPH surgery has the disadvantages of unknown long-term efficacy, external hemorrhoid retraction insufficiency, re-prolapse, perianal edema, and pain for many patients (13,14). In addition, patients with mixed hemorrhoids expect improvement of symptoms and anal cosmetics post-operatively (2). Therefore, for patients with mixed hemorrhoids, especially circumferential mixed hemorrhoids, it is important to explore the surgical methods for complete resolution of the symptoms, less trauma, greater efficacy, fewer complications, a lower recurrence rate, as well as maintenance of normal anal anatomy and function. The present study was designed to determine the effect of segmental and plastic hemorrhoidectomy (SPH) for the treatment of mixed hemorrhoids with predominant circumferential external hemorrhoids.

## Material and Methods

### Patients

A multicenter, single-blinded, randomized controlled trial was conducted. A total of 300 patients with mixed hemorrhoids and a predominance of circumferential external hemorrhoids were randomly selected from the following sites between July 2011 and July 2013: 1) Hemorrhoids and Fistula Center of Integrated Traditional Chinese and Western Medicine, West China Hospital of Sichuan University (n=100); 2) Department of Anoproctology, the Affiliated Hospital of Chengdu University of Traditional Chinese Medicine (n=100); and 3) Department of Anoproctology, Chengdu Hospital of Traditional Chinese and Western Medicine (n=100). SPSS statistical software (12th edition; IBM, USA) was used to design the random number table and the patients were randomly divided into an experimental group (n=150) that underwent SPH and a control group (n=150) that underwent Milligan-Morgan hemorrhoidectomy. Before the study, detailed surgical specifications and standard operating procedures were formulated. A senior attending surgeon with experience of more than 10 years was selected at each center to perform the operations. Each selected surgeon also received specific training in order to reduce the result bias. All patients provided signed informed consent for participation in the study and approval for the study was obtained from the Ethics Committee of West China Hospital of Sichuan University, China. The demographic data, including gender, age, weight, and height, are shown in Table 1.

**Table 1:** Demographic data for the two groups.

Items	Experimental group (n=150)	Control group (n=150)	P value
Gender, male/female	71/79	74/76	>0.05
Age, mean±SD (range), years	47.05±13.75 (20-70)	46.10±12.70 (22-68)	>0.05
Weight, mean±SD (range), kg	61.96±12.95 (44.2-85.4)	61.76±12.87 (47.3-81.4)	>0.05
Height, mean±SD (range), cm	163.73±7.07 (150-179)	162.05±8.75 (148-185)	>0.05

The *t*-test and χ^2^ test were used for statistical analysis.

### Inclusion criteria

The inclusion criteria for the study were as follows: 1) 20–70 years of age; 2) patients conformed to the diagnostic criteria of mixed hemorrhoids (12,15); 3) the internal hemorrhoids were grade III or above; 4) routine blood and urine testing, liver and kidney function testing, and psychological testing were normal; and 4) there were no severe heart or pulmonary diseases, no dysentery or severe diarrhea, no systemic diseases, and no other abnormal anorectal conditions, such as anal fissures, anal fistulas, and anorectal tumors.

### Operative method

All patients were asked not to consume a solid diet after 8:00 p.m. the night before surgery. Epidural anesthesia was used and the patient was placed in the lithotomy position.

#### Experimental group

The anus was disinfected and sufficiently dilated. A radial incision was made in the annular protrusion of the external hemorrhoids, with the incision extending approximately 0.5 cm to the dentate line and 0.5 cm distal to the hemorrhoids. The skin was cut, followed by subcutaneous expansion of the vascular plexus. Thromboses and hyperplastic lesions in the distal dentate line were isolated and dissected. In addition, an auxiliary radial incision was made to expose and remove the external hemorrhoid lesions. The flap between the two incisions was clamped transversely approximately 1.0 cm below the dentate line along the inter-sphincteric groove plane, insuring that the anal canal flap and tension were moderate. This step is key for the following reasons: 1) proximity to the dentate line can damage the sensitive anal transitional zone epithelium, consequently leading to a disturbance in anal function post-operatively and pain and 2) proximity to the anus is not conducive to shaping the perianal skin flap for reconstruction of the anus. The flap was removed under the clamp edge at the same time the incision was sutured transversely. A subcutaneous suture was placed proximal to the radial incision at a depth of the superficial internal sphincter, making the anal skin and subcutaneous tissue fixed in the internal sphincter. Based on the distribution, morphology, and number of internal hemorrhoids, different surgical methods were used. Ligation and injection was adopted for non-circumferential internal hemorrhoids and PPH surgery was used for circumferential internal hemorrhoids. Analgesic liquid (5 mL of 0.5% bupivacaine + 4 mL of 2% lidocaine + 1 mL of 1% methylene blue) was used post-operatively. During the operation, the lower edge of internal sphincter in the non-maternal hemorrhoid area should be cut open to dilate the anus. If the anus is not dilated adequately, anus stenosis may be caused by scarring after operation.

#### Control group

After the anus was disinfected, the top of the external hemorrhoid was clamped and gently pulled outward for exposure of the internal hemorrhoids. A V-shape incision was made in the skin on both sides of the external hemorrhoids using surgical scissors, followed by stripping the hemorrhoids between the subcutaneous vein and sphincter muscle to the dentate line (0.2 cm). The stripped external hemorrhoids and the basal part of the internal hemorrhoids were clamped, followed by suturing and ligaturing the root of the internal hemorrhoids with 0 silk suture, then dissecting the partial internal hemorrhoids. The same method was used to strip, suture, and ligature the other hemorrhoids. The skin and mucous bridge should be kept between the suture-ligature points. Under an anoscope, 3∼5 mL of Xiao Zhi Ling solution (containing alum, tannic acid, and low molecular dextran; Ji’an Yisheng Pharmaceutical Company, Jiling Provience, China) was injected submucosally into the area of the artery supplying the suture-ligatured hemorrhoid to achieve hemostasis. The post-operative analgesic liquid (same as above) was injected during closure of the wound.

#### Post-operative management

All patients were given a liquid diet 2 h post-operatively and intravenous antibiotics for prophylaxis. All of the patients were advanced to a normal diet the second day post-operatively. Bathing was permitted after the first defecation. The dressing was changed in the morning and evening or after defecation.

### Evaluation of the therapeutic effect and anal function

The therapeutic effect was assessed based on the item of curative effect evaluation of mixed hemorrhoids in the Criteria for Diagnosis and Curative Effect of Diseases and Syndromes of Traditional Chinese Medicine issued by the State Administration of Traditional Chinese Medicine in 1994. The therapeutic effects are divided into three grades: cure, effective, and ineffective. Briefly, resolution of symptoms and hemorrhoids was considered a cure; improvement in symptoms and a decrease in the size of the hemorrhoids was considered effective; and no change in symptoms and hemorrhoids was considered ineffective. The time to wound healing and recovery to normal activity in days were determined and recorded.

Anal functions 6, 12, and 18 months post-operatively were assessed as described previously (16) with the following criteria: good control of the stool, intestinal liquid, and intestinal gas was considered normal; no control of loose stool, intestinal liquid, and intestinal gas, soiling of the underwear, or a moist anus was considered partial incontinence; and no control of stool was considered complete incontinence. Anal stenosis or constriction was evaluated using yes-and-no criteria.

Anal smoothness 6, 12, and 18 months post-operatively was evaluated according to the following criteria: smoothness of the local anus without protrusions was considered grade I; <3 slight protrusions in the local anus without related symptoms was considered grade II; and large protrusions or numerous slight protrusions with related symptoms was considered grade III.

Patient satisfaction was determined by questionnaire and categorized into dissatisfied, ordinary, and satisfied.

### Evaluation of post-operative complications

Pain was evaluated using a visual analogue scale (VAS) with a score of 0-10 during defecation for 7 consecutive days post-operatively, in which 0 represented no pain and 10 represented severe, intolerable pain. Uroschesis was evaluated as yes-or-no. Hematochezia was evaluated using a score of 0–3 within 1 month post-operatively, in which 0 represented no hematochezia, and 1, 2, and 3 represented blood visible in the stool, dripping of blood, and flowing of blood, respectively. Edema was evaluated using a score of 0–4 post-operatively, in which 0 represented no edema, and 1, 2, 3, and 4 represent edema involving 25, 50, 75, and 100% of the anal edge, respectively.

### Statistical analysis

SPSS 19.1 software (IBM) was used to analyze the data. Quantitative data, such as age and operative time, are reported as means±SD, and a *t*-test was used for comparisons among groups. Enumerated data, such as gender, stage, and some post-operative complications, were analyzed using a χ^2^ test. Postoperative pain score, edema, and hematochezia are reported as median (minimum and maximum) and were evaluated by the rank-sum test. P<0.05 indicated a statistically significant difference.

## Results

### Demographic data

As shown in Table 1, no differences in gender, age, weight, and height existed between the two groups.

### Comparison of the therapeutic effect between the two groups

**Figure 1:**
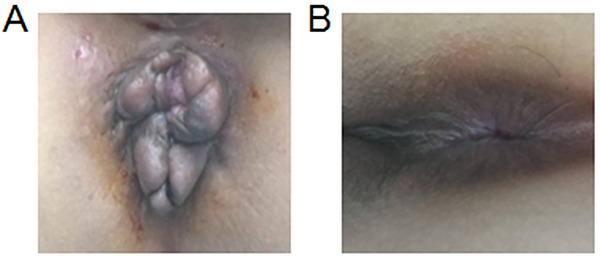
Therapeutic effect of segmental resection combined with anus plasty. **A**, Representative circumferential mixed hemorrhoids pre-operatively with grade IV internal hemorrhoids and symptoms of prolapse. **B**, One month post-operatively, the prolapse symptoms were resolved and the hemorrhoids nearly disappeared with grade I anal smoothness.

Improvement in hemorrhoid symptoms did not differ between the two groups (Table 2); however, compared with the control group, patients in the experimental group had a shorter time for wound healing and recovery to normal activity. The follow-up evaluations 6, 12, and 18 months post-operatively showed that anal function in all patients in the experimental group was normal and the stool, intestinal liquid, and gas were well-controlled; however, 6, 8, and 14 patients in the control group had anal dampness, itching, and poor control of intestinal liquid at the 6-, 12-, and 18-month follow-up evaluations, respectively. No anal stenosis was noted in either group 6–18 months post-operatively. The appearance of the anus revealed grade I, II, and III smoothness in 147 (98.0%), 3 (2.0%), and 0 (0%) patients in the experimental group, respectively (Figure 1), 6 months post-operatively. In contrast, anal smoothness was grade I, II, and III in 113 (75.3%), 20 (13.3%), and 17 (11.3%) patients in the control group, respectively (Figure 1), 6 months post-operatively. Similarities in anal smoothness existed 12 and 18 months post-operatively. Overall, anal function and smoothness in the experimental group was better than the control group. Twelve months post-operatively, 0 (%), 98 (65.3%), and 52 (34.7%) patients were dissatisfied, ordinary, and satisfied, respectively, in the experimental group; however, 8 (5.3%), 101 (67.4%), and 41 (27.3%) patients were dissatisfied, ordinary, and satisfied, respectively, in the control group (Table 3). Both groups of patients had similar levels of satisfaction 18 months post-operatively.

**Table 2:** Comparison of therapeutic effect between the two groups.

Items	Experimental group (n=150)	Control group (n=150)	P value
Efficacy, n (%)			>0.05
Cured	146 (97.3)	144 (96.0)	
Effective	4 (2.7)	6 (4.0)	
Invalid	0 (0)	0 (0)	
Wound healing time, days	12.5±0.7	15.7±1.3	<0.05
Recovery time to normal activity, days	14.7±1.2	18.5±2.7	<0.05
Anal function 6 month after operation, n (%)			<0.05
Normal	150 (100)	144 (96.0)	
Partial incontinence	0 (0)	6 (4.0)	
Complete incontinence	0 (0)	0 (0)	
Anal function 12 month after operation, n (%)			0.004
Normal	150 (100)	142 (94.7)	
Partial incontinence	0 (0)	8 (5.3)	
Complete incontinence	0 (0)	0 (0)	
Anal function 18 month after operation, n (%)			<0.001
Normal	150 (100)	136 (90.7)	
Partial incontinence	0 (0)	14 (9.3)	
Complete incontinence	0 (0)	0 (0)	
Anal stenosis or sense of contraction 6, 12, and 18 months after operation, n (%)	0 (0)	0 (0)	
Anal smoothness 6 months after operation, n (%)			<0.05
I	147 (98.0)	113 (75.3)	
II	3 (2.0)	20 (13.3)	
III	0 (0)	17 (11.3)	
Anal smoothness 12 months after operation, n (%)			<0.001
I	145 (96.7)	122 (81.3)	
II	5 (3.3)	15 (10.0)	
III	0 (0)	13 (8.7)	
Anal smoothness 18 months after operation, n (%)			<0.001
I	143 (95.3)	117 (78.0)	
II	7 (4.7)	20 (13.3)	
III	0 (0)	13 (8.7)	

Data are reported as means±SD or number and percentage.

**Table 3:** Comparison of postoperative patient satisfaction between the two groups.

Items	Experimental group(n=150)	Control group(n=150)	P value
12 months after operation, n (%)			0.046
Unsatisfied	0 (0)	8 (5.3)	
Ordinary	98 (63.5)	101 (67.4)	
Satisfied	52 (34.7)	41 (27.3)	
18 months after operation, n (%)			0.047
Unsatisfied	0 (0)	10 (6.7)	
Ordinary	102 (68.0)	102 (68.0)	
Satisfied	48 (32.0)	38 (25.3)	

Data are reported as number and percentage.

### Comparison of post-operative complications between the two groups

As shown in Table 4, the number of patients with uroschesis and the VAS for hematochezia 1 month post-operatively were comparable between the two groups; however, the VAS for dyschezia 7 consecutive days post-operatively and edema in the experimental group was less than the control group.

**Table 4:** Comparison of postoperative complications between the two groups.

Items	Experimental group (n=150)	Control group(n=150)	P value
Defecation pain 7 consecutive days after operation, VAS	3 (0-10)	5 (0-10)	<0.05
Edema, VAS	1 (0-4)	3 (0-4)	<0.05
Uroschesis, n (%)	21 (14.0)	24 (16.0)	>0.05
Hematochezia 1 month after operation, VAS	1 (0-3)	1 (0-3)	>0.05

VAS: visual analog scale. Data are reported as median (minimum and maximum) and evaluated by the rank-sum test.

## Discussion

Surgical methods for mixed hemorrhoids include the external piles-excision and internal piles-ligature operation, namely the classic Milligan-Morgan hemorrhoidectomy, and the PPH. Watson et al. (17) conducted a randomized controlled study of PPH and traditional surgery (TS) and showed that patients who received stapled hemorrhoidopexy (SH) had less short-term pain; however, after 6 weeks, recurrence rates, symptoms, re-interventions, and quality-of-life measures all favored TS. In addition, TS is cheaper. As part of a tailored management plan for hemorrhoids, TS should be considered over SH as the surgical treatment of choice for hemorrhoids refractory to clinic-based interventions. A long-term follow-up study showed that there was no significant difference between PPH and Ferguson hemorrhoidectomy in patient satisfaction, improvement of hemorrhoid symptoms, quality of life, and anal function. In recent years, an increasing number of surgeons have used hemorrhoid artery ligation to treat stage III hemorrhoids (18). Transanal hemorrhoidal dearterialization (THD) offers a safe surgery with significantly less postoperative bleeding compared with SH. However, SH had a lower recurrence rate compared with THD. Trenti et al. (19) conducted a randomized controlled study of THD and TS, suggesting that THD is not inferior to conventional excisional hemorrhoidectomy for advanced hemorrhoidal disease in terms of postoperative complications and long-term recurrence of symptoms.

Hemorrhoids are a completely benign condition, yet the treatment should offer minimal invasion and the highest degree of safety and recovery time for the patient. In addition, with improvements in living standards, more patients with mixed hemorrhoids focus on improvement of symptoms and have high expectations for anal esthetics post-operatively (20). Furthermore, to simply improve the symptoms of hemorrhoids rather than to completely resolve the underlying pathologic problem may lead to recurrences or aggravation of mixed hemorrhoids, thus affecting the quality of life. Therefore, for patients with mixed hemorrhoids, especially circumferential mixed hemorrhoids, it is important to offer the optimal surgical method for complete elimination of the symptoms, with low trauma, few complications, and a low recurrence rate, maintaining normal anal anatomy and function.

The present study showed that although no differences in cure and efficacy rates on mixed hemorrhoids between the two groups were noted, patients in the experimental group had a shorter time for wound healing and recovery to normal activity compared with the control group, suggesting that SPH has advantages compared with the classic Milligan-Morgan hemorrhoidectomy. Follow-up evaluations 6, 12, and 18 months post-operatively showed that anal function of all patients in the experimental group was better than the control group at the corresponding times, which might be explained by the fact that the surgery completely maintained the dentate line and the anorectal transitional zone epithelium, consequently preserving anal continence and fine sensory function. Overall, anal smoothness in the experimental group was better than the control group, which might be explained by the fact that the external part of hemorrhoids was segmented followed by discontinuous and transverse resection and suture of the incision, consequently removing the excess perianal skin tags and reducing the risk of recurrence. In addition, overall satisfaction in the experimental group was better than the control group, which might be attributed to fewer complications, a lower recurrence rate, and better anal appearance during long-term observation due to the maximal retention of normal tissue of the anus and resection of the lesion of the anus.

With respect to the post-operative complications, the VAS for dyschezia 7 consecutive days post-operatively and edema in the experimental group was less than the control group, because cutting part of the lower margin of the inner sphincter, dilating the anus, and relieving spasm during SPH decreased the anal resting pressure, thus decreasing the internal sphincter spasm stimulated by pain and alleviating the edema due to the disorders of blood and lymph circulation.

In summary, SPH had the advantages of efficacy, fewer complications, better protection of anal function, and an improved cosmetic effect, which is worthy of clinical application. Although there are many advantages, SPH had no superiority with respect to cure and effective rates for hemorrhage and complications, such as uroschesis and the VAS for hematochezia 1 month post-operatively compared to the Milligan-Morgan hemorrhoidectomy. Moreover, the operation time of SPH is relatively long. An SPH operation takes about 1 to 2 h. It requires high professional skills of the surgical team. If the operation is improper, there is a risk of anal stenosis. As a novel method, there may be potential risks that have not been identified. Future studies are therefore warranted to fully evaluate the clinical value of this surgical method.
